# AMP-activated protein kinase α2 contributes to acute and chronic hyperuricemic nephropathy via renal urate deposition in a mouse model

**DOI:** 10.1186/s40001-022-00800-1

**Published:** 2022-09-10

**Authors:** Chen Yang, Hong-yong Su, Ning An, Hong-luan Wu, Xiao-yan Guo, Zhi-hang Li, Xiao-cui Chen, Shao-ping Zhu, Dan Wu, Hui-yuan Li, Qing-jun Pan, Dong Liang, Hua-feng Liu

**Affiliations:** 1 Key Laboratory of Prevention and Management of Chronic Kidney Disease of Zhanjiang City, Institute of Nephrology, Affiliated Hospital of Guangdong Medical University, 57 Renmin Road, Zhanjiang, 524001 Guangdong China; 2 Guangdong Provincial Key Laboratory of Autophagy and Major Chronic Non-Communicable Diseases, Zhanjiang, 524001 Guangdong China; 3 Laboratory Animal Center, Guangdong Medical University, Zhanjiang, 524001 Guangdong China

**Keywords:** Hyperuricemia, Renal inflammation, Renal fibrosis, Urate deposition, AMPK α2

## Abstract

Hyperuricemia can induce acute and chronic kidney damage, but the pathological mechanism remains unclear. The potential role of AMP-activated protein kinase (AMPK) α2 in hyperuricemia-induced renal injury was investigated in this study. Acute and chronic hyperuricemic nephropathy was induced by administering intraperitoneal injections of uric acid and oxonic acid to AMPK α2 knockout and wild-type mice. Changes in renal function, histopathology, inflammatory cell infiltration, renal interstitial fibrosis, and urate deposition were analyzed. In both acute and chronic hyperuricemic nephropathy mouse models, knockout of AMPK α2 significantly reduced serum creatinine levels and renal pathological changes. The tubular expression of kidney injury molecule-1 was also reduced in hyperuricemic nephropathy mice deficient in AMPK α2. In addition, knockout of AMPK α2 significantly suppressed the infiltration of renal macrophages and progression of renal interstitial fibrosis in mice with chronic hyperuricemic nephropathy. Knockout of AMPK α2 reduced renal urate crystal deposition, probably through increasing the expression of the uric acid transporter, multidrug resistance protein 4. In summary, AMPK α2 is involved in acute and chronic hyperuricemia-induced kidney injury and may be associated with increased urate crystal deposition in the kidney.

## Introduction

Uric acid (UA) is the end product of purine metabolism in humans. It is not a bypass waste, but rather a powerful antioxidant that protects humans from oxygen radicals [18]. Hyperuricemia occurs when there is an excessive level of UA in the blood due to increased production or decreased excretion. It is clinically defined as a serum UA level ≥ 416 μmol/L in men and ≥ 339 μmol/L in premenopausal women, measured twice on different days while following a normal purine diet [7]. Owing to the changes in lifestyle and an increase in the elderly population, the incidence of hyperuricemia in China has risen rapidly in the last two decades, reaching nearly 16.4% [9]. Growing evidence has demonstrated that hyperuricemia is closely associated with various human diseases including renal diseases. Moreover, 71% of gout patients have chronic kidney disease (CKD) stage 2 or higher, and 24% have kidney stones [11]. Hyperuricemia becomes an independent risk factor for diabetic nephropathy, acute kidney injury (AKI), chronic kidney disease (CKD), and end-stage renal disease (ESRD) [3].

In recent years, increased attention has been given to the kidney damage caused by hyperuricemia, but the pathological mechanisms remain unknown [17]. Although asymptomatic hyperuricemia was reported in CKD, soluble UA seems to promote renal inflammation via the activation of NLRP3 inflammasome and the synthesis of IL-1β [5], these data were challenged recently [10, 15]. Hyperuricemia has been shown to cause kidney injury directly through urate deposition in the kidneys, resulting in kidney stones, tubular obstruction, tubule necrosis, macrophage-mediated interstitial nephritis, renal fibrosis, and finally, renal function loss [15]. As a result, increased attention is required to avoid urate crystal formation and deposition in the kidneys.

AMP-activated protein kinase (AMPK), widely expressed in eukaryotes, mainly regulates cellular energy metabolism. AMPK is composed of an alpha catalytic subunit (α1 or α2), a non-catalytic beta (β1 or β2), and gamma subunits (γ) [21]. Drugs activating AMPK, such as metformin, are renoprotective via autophagy activation, anti-aging, anti-oxidative stress, anti-endoplasmic reticulum stress (ER-stress), anti-inflammatory, and anti-fibrosis properties [13]. AMPK may also play a key role in hyperuricemia and hyperuricemia-induced kidney injury, however, its specific role is still controversial. On the one hand, the AMPK activator drug, metformin, was explored to reduce the frequency of gout attacks in gout patients [19]. Through two prospective experiments, Barskova, VG et al. demonstrated that metformin significantly reduced serum UA levels and insulin resistance in patients with gout, and speculated that metformin may reduce UA production by inhibiting fatty acid synthesis [1]. Krzystek-korpacka et al. found that both metformin and weight loss could significantly reduce the serum UA levels of juvenile diabetic patients [6]. On the other hand, the opposite result also existed. Some studies reported that metformin causes a significant increase in serum UA and creatinine levels in Zucker Diabetic Fatty type 2 diabetic rat models [12]. Yarovoi SK et al. also demonstrated that metformin causes urine acidification in diabetic patients, which in turn promotes urate crystal deposition in the kidneys [24].

In summary, the role of AMPK in hyperuricemic nephropathy (HN) is not fully understood. To date, nearly all experiments that investigated the functional role of AMPK in hyperuricemia-induced kidney injury used drugs rather than genetically modulated animals. Thus, in our study, we investigated whether AMPK enhances or reduces acute and chronic kidney damage caused by hyperuricemia using AMPK α2 knockout mice. Our findings provide laboratory evidence on the use of AMPK activators in the treatment of patients with hyperuricemia.

## Materials and methods

### Reagents and antibodies

The Creatinine Assay kit, Blood Urea Nitrogen (BUN) Assay kit, and Uric acid Test Kit were purchased from Nanjing Jiancheng Bioengineering Institute (Nanjing, China). Uric acid sodium salt (U2875), oxonic acid potassium salt (156,124), and sodium carboxymethyl cellulose (CMC-Na, 419,273) were purchased from Sigma (St. Louis, USA). Anti-AMPK α2 antibody (ab3760), anti-collagen I antibody, anti-α-smooth muscle actin (α-SMA) antibody, anti-MRP4 antibody (ab180712), and anti-kidney injury molecule-1 (KIM-1) antibody were purchased from R&D systems (Minnesota, USA). The 3,30-diaminobenzidine (DAB) and anti-glyceraldehyde-3-phosphate dehydrogenase (GAPDH) monoclonal antibody (AF1186) was purchased from the Beyotime Institute of Biotechnology (Shanghai, China).

### Animal

The AMPK α2^−/−^ mice [20] (originally generated in Dr. Benoit Viollet’s lab) were donated by Prof. Ying Zhang of Beijing Sport University and were bred in the Laboratory Animal Services Center of Guangdong Medical University. The genotyping was performed as described in the study of Li et al. [8]. The mice were kept in a pathogen-free room with a temperature of 22 ± 2 °C, 50 ± 10% relative humidity, and a 12-h light/dark cycle. Food and water were provided ad libitum. All animal experiments were approved by the Laboratory Animal Services Center of Guangdong Medical University (Zhanjiang, China) (No. GDY1902138) and performed according to the guidelines of Animal Welfare and Ethics of the Institutional Animal Care and Use Committee.

### Genotyping

AMPK α2^−/−^ genotype was confirmed by PCR analysis. In brief, DNA was extracted from mouse tail tissue. PCR was performed by 2×Hieff^®^ PCR Master Mix (Yeasen Biotechnology, Shanghai, China), using the following primers: AMPK α2 wild-type (forward: 5′-GCTTAGCACGTTACCCTGGATGG-3′, reverse: 5′-GTTATCAGCCCAACTAATTACAC-3′) and AMPK α2^−/−^ (forward: 5’-GCTTAGCACGTTACCCTGGATGG-3′, reverse: 5′-GCATTGAACCACAGTCCTTCCTC-3′). The DNA products were placed on a 2% agarose gel for electrophoresis, and images were acquired by using the Azure C500. Only DNA band near 200 bp indicates wild-type homozygote, and only DNA band near 600 bp indicates knockout homozygote. PCR analysis shows both DNA bands of 200 bp and 600 bp indicating heterozygote genotype.

### Acute and chronic HN

For the acute HN experiment, 8-week-old AMPK α2^+/+^ (wild-type, WT) and AMPK α2^−/−^ (knockout, KO) mice weighing 18–25 g were divided into four groups: the WT control group (WT + CON), the KO control group (KO + CON), the WT acute HN group (WT + Acute HN) and the KO acute HN group (KO + Acute HN). The acute HN model was induced by intraperitoneal injections with UA (200 mg/kg) and oxonic acid (OA) (150 mg/kg) three times with an interval of 12 h. Water intake was restricted for 24 h after the first injection to facilitate hyperuricemia [23]. The control mice were injected with an equal volume of vehicle (saline and 0.5% CMC-Na solution). All mice were anesthetized by intraperitoneal injection with an overdose of sodium pentobarbital (100 mg/kg) and killed 24 h after the last injection, and the serum and kidneys were harvested for analysis.

For the chronic HN experiment, same-aged mice were also divided into four groups: the WT control group (WT + CON), the KO control group (KO + CON), and the WT chronic HN group (WT + Chronic HN), and the KO chronic HN group (KO + Chronic HN). Chronic HN models were induced by administering an intraperitoneal injection of UA (200 mg/kg) and OA (150 mg/kg) once a day for 3 weeks. Water was provided ad libitum. Mice under the control group were injected with an equal dose of vehicle (saline and 0.5% CMC-Na solution) [16]. All mice were anesthetized by intraperitoneal injection with an overdose of sodium pentobarbital (100 mg/kg) and killed 24 h after the 3 weeks of injections, and their serum and kidneys were harvested for analysis.

### Renal function evaluation

The serum creatinine (Scr) and BUN levels were detected using the colorimetric method according to the manufacturer’s instructions.

### Histopathology

The kidneys were harvested and fixed in 4% paraformaldehyde (pH 7.4), then dehydrated, and embedded in paraffin. A 3-μm section was stained with either a periodic acid–Schiff (PAS), Masson, or Sirius red stain. Two pathologists scored the kidney injury based on PAS staining in a blinded manner using the following criteria: (1) dilatation of renal tubules: 0–5% area of section (0 point), 5–25% area of section (1 point), 25–50% area of section (2 points), 50–75% area of section (3 points), > 75% area of section (4 points); (2) shedding of brush border: 0–5% area of section (0 point), 5–25% area of section (1 point), 25–50% area of section (2 points), 50–75% area of section (3 points), > 75% area of section (4 points); (3) vacuolar degeneration of tubular epithelial cells (TECs): 0–5% area of section (0 point), 5–25% area of section (1 point), 25–50% area of section (2 points), 50–75% area of section (3 points), > 75% area of section (4 points); and (4) cast: no cast in the section (0 point), 1 cast in the section (1 point), 2 cast in the section (2 points), 3 cast in the section (3 points), more cast in the section (4 points). The total score was the sum of all scores in the renal section samples.

To detect urate crystal deposition, kidneys were fixed and dehydrated in absolute ethyl alcohol, embedded in paraffin. A 5-μm section was stained with eosin stain for either light or polarized light microscopy. The positive areas (%) for urate crystal deposition were measured using Image J software (NIH, USA).

### Immunohistochemistry

The expression of collagen I, α-smooth muscle actin (α-SMA), and kidney injury molecule-1 (KIM-1) in the kidneys was determined by immunohistochemistry (IHC). The paraffin-embedded kidney sections were deparaffinized and rehydrated. After epitope retrieval and blockade of endogenous peroxidase, the sections were incubated with primary and HRP-conjugated secondary antibodies, followed by DAB immunostaining and hematoxylin counterstaining. The area (%) of positive staining was measured using Image J software (NIH, USA).

### Immunofluorescence

The macrophage infiltration was evaluated by the immunofluorescence (IF) of F4/80. In brief, the frozen section of the kidneys was blocked with 5% bovine serum albumin and sequentially incubated with rat anti-mouse F4/80 antibody (MCA497GA) and Alexa-594-conjugated donkey anti-rat IgG. The nuclei were then visualized with 4′,6-diamidino-2-phenylindole (DAPI, D1306, Invitrogen). The fluorescence signals were visualized using a confocal microscope (Olympus FV3000, Japan), and positively stained cells per field, in 10 randomly selected fields, were counted for quantification.

### Western blot

The expression of target proteins such as AMPK α2 and MRP4 in the kidney was determined using the western blot (WB). All the proteins were transformed from sodium dodecyl sulfate–polyacrylamide gel electrophoresis to sodium dodecyl sulfate–polyacrylamide gel electrophoresis, followed by incubation with the primary and HRP-conjugated secondary antibodies. Glycolytic glyceraldehyde-3-phosphate dehydrogenase (GAPDH) was used as a loading control. The integrated optical density and the area of protein bands were quantified and analyzed with Image J software (National Institutes of Health, Maryland, USA).

### Statistical analysis

All the experimental data are shown as means ± standard error of the mean (SEM). For comparison among multiple groups, a one-way analysis of variance was used followed by Tukey’s post hoc tests. A *P* value < 0.05 was considered a statistically significant difference in this study. Data analysis was performed, and graphics were created using GraphPad Prism 5 (GraphPad Software, San Diego, CA, USA).

## Results

### AMPK α2 exacerbated kidney injury in a mouse model of acute HN

**Fig. 1:**
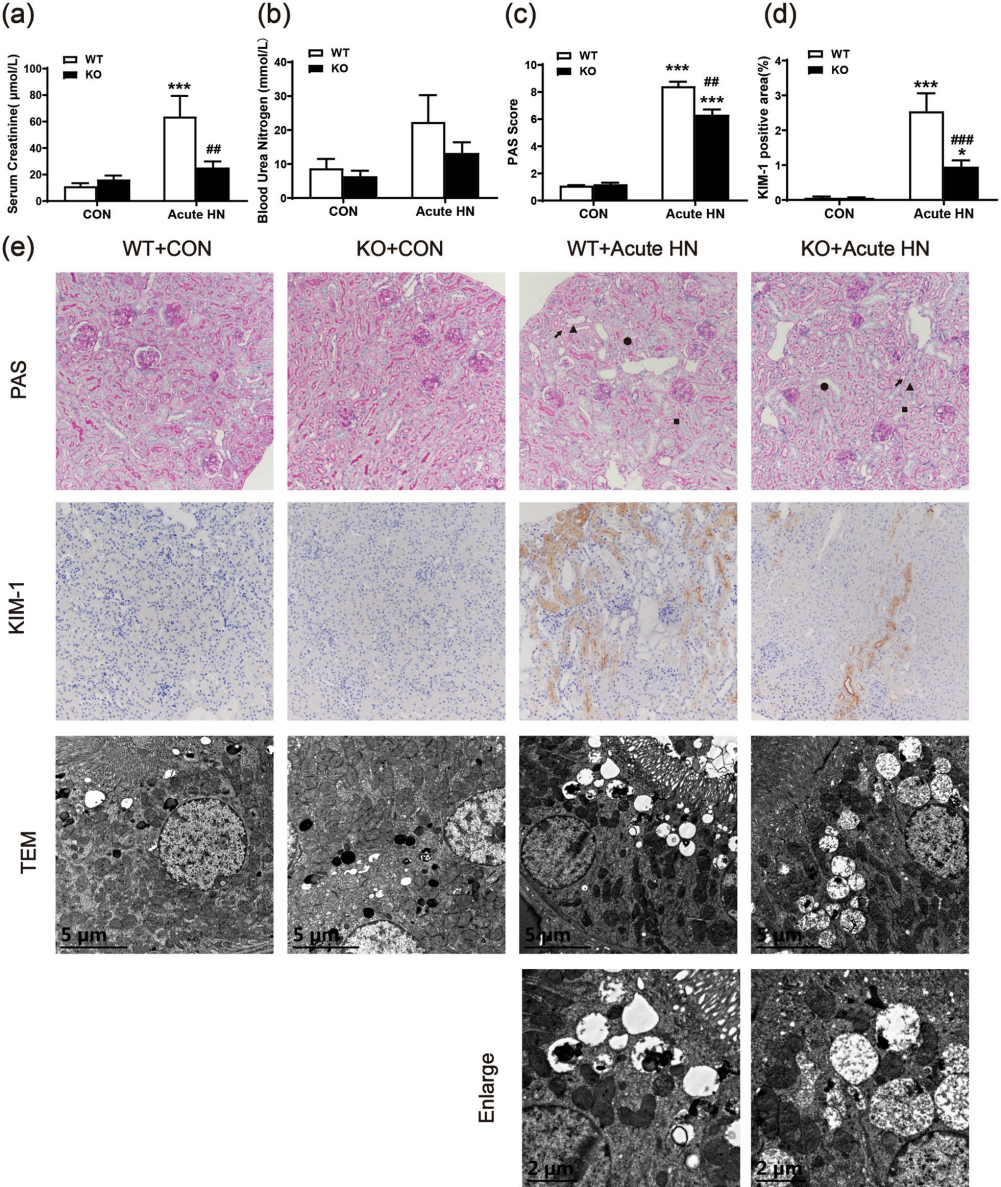
AMPK α2 exacerbated kidney injury in a mouse model of acute HN. **a** Serum creatinine. **b** Blood urea nitrogen. **c** A pathological score of tubular injury in the kidneys. **d** Quantitative analysis of KIM-1-positive area in the kidneys. **e** Representative images of PAS staining (arrow: renal tubules dilatation, triangle: brush border shedding, square: tubular vacuolization, round: casts formation), KIM-1 immunohistochemical staining, and transmission electron microscopy. Each bar represents the mean ± SEM. Versus CON, *P < 0.05 and ***P < 0.001; versus WT, ^##^*P* < 0.01 and ^###^*P* < 0.001

First, we tested the functional role of AMPK α2 in a mouse model with acute HN. As shown in Fig. 1a, in contrast to that of the control mice, Scr levels were significantly increased in mice that were given UA and OA intraperitoneal injections. Surprisingly, AMPK α2 knockout notably downregulated the level of Scr in acute HN mice (Fig. 1a). The level of serum BUN displayed a similar trend as Scr between the four groups but with no significant difference (Fig. 1b). PAS staining was performed to analyze and quantify the pathological changes in the kidneys. The specific morphological changes of kidneys were developed in acute HN mice, including renal tubules dilatation, brush border shedding, tubular vacuolization, and cast formation. In contrast, AMPK α2 knockout remarkably attenuated these pathological changes that developed in acute HN mice (Fig. 1c and e). Consistent with these findings, elevated renal expression of KIM-1, a marker of tubular injury, was also downregulated in acute HN mice deficient in AMPK α2 (Fig. 1d and e). Finally, we performed transmission electron microscopy to further analyze the pathological changes (Fig. 1e). In comparison to the control group, acute HN mice had obvious renal tubular dilatation within cast formation, which were mainly cell debris. In addition, more pinocytosis vesicles, which fused with lysosomes, were found in the renal tubular epithelial cells of the acute HN mice, while the brush border of the renal TECs was significantly shed. All these pathological changes were reduced in acute HN mice deficient in AMPK α2. These findings suggested that AMPK α2 exacerbated kidney injury in a mouse model of acute HN.

### AMPK α2 exacerbated kidney injury in a mouse model of chronic HN

**Fig. 2:**
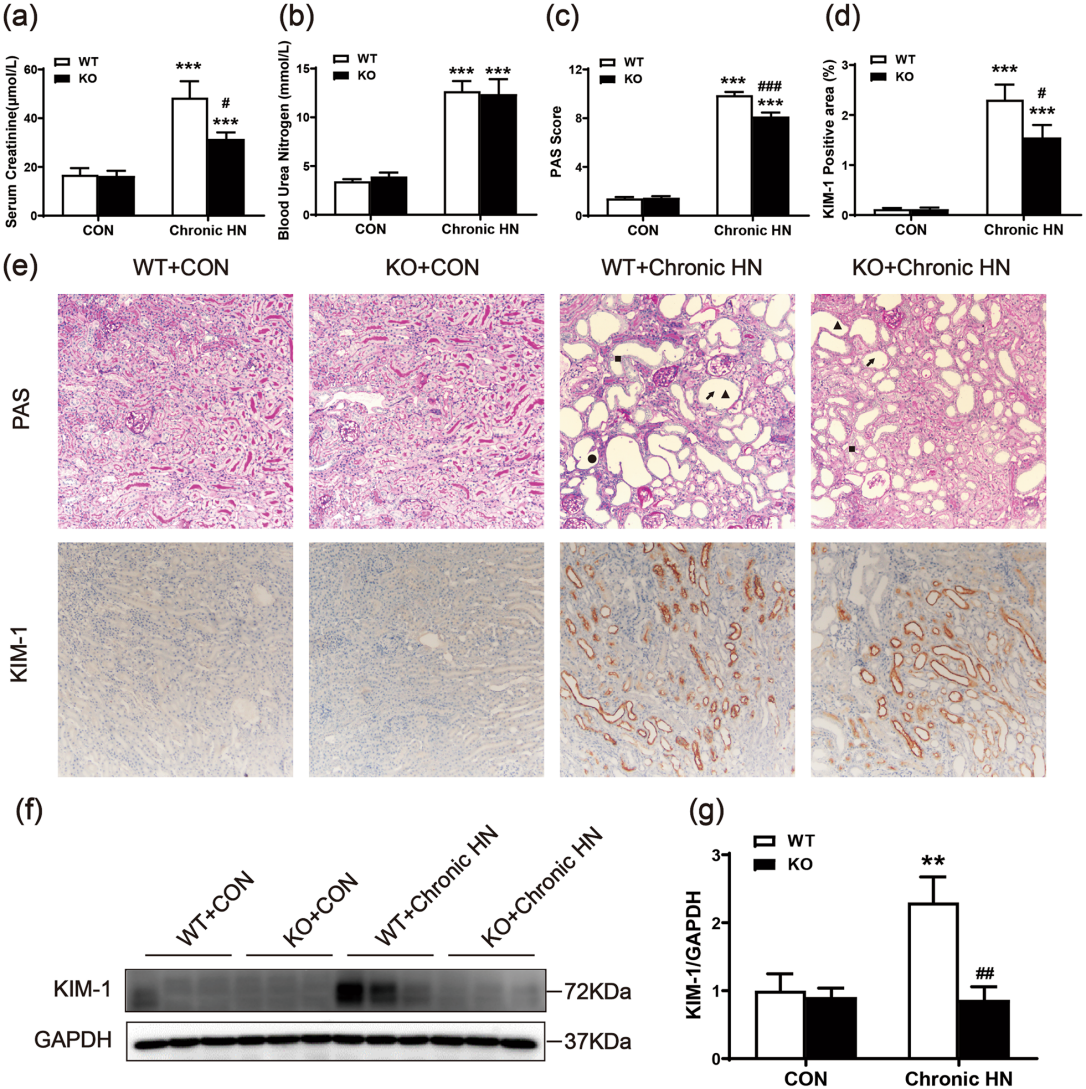
AMPK α2 exacerbated kidney injury in a mouse model of chronic HN. **a** Serum creatinine. **b** Blood urea nitrogen. **c** A pathological score of tubular injury in the kidneys. **d** Quantitative analysis of KIM-1-positive area in the kidneys. **e** Representative images of PAS staining (arrow: renal tubules dilatation, triangle: brush border shedding, square: tubular vacuolization, round: casts formation) and KIM-1 immunohistochemical staining. **f**, **g** Western blot analysis of KIM-1 expression in the kidneys. Each bar represents the mean ± SEM. Versus CON, ***P < 0.001; versus WT, ^#^*P* < 0.05, ^##^*P* < 0.01 and ^###^*P* < 0.001

Hyperuricemia not only induces AKI, but also causes chronic renal lesions. Next, we tested the role of AMPK α2 in a mouse model with chronic HN. Compared to the control mice, Scr and BUN were significantly increased in chronic HN mice, while AMPK α2 knockout reduced Scr levels but only had a little effect on BUN levels in chronic HN mice (Fig. 2a and b). PAS staining revealed that AMPK α2 knockout also attenuated the pathological changes that develop in chronic HN mice (Fig. 2c and e). Moreover, the renal expression of KIM-1 also increased in chronic HN mice, while the level of tubular KIM-1 was notably downregulated in the chronic HN mice deficient in AMPK α2 (Fig. 2d-2g).

### AMPK α2 exacerbated renal fibrosis and macrophage infiltration in a mouse model of chronic HN

**Fig. 3:**
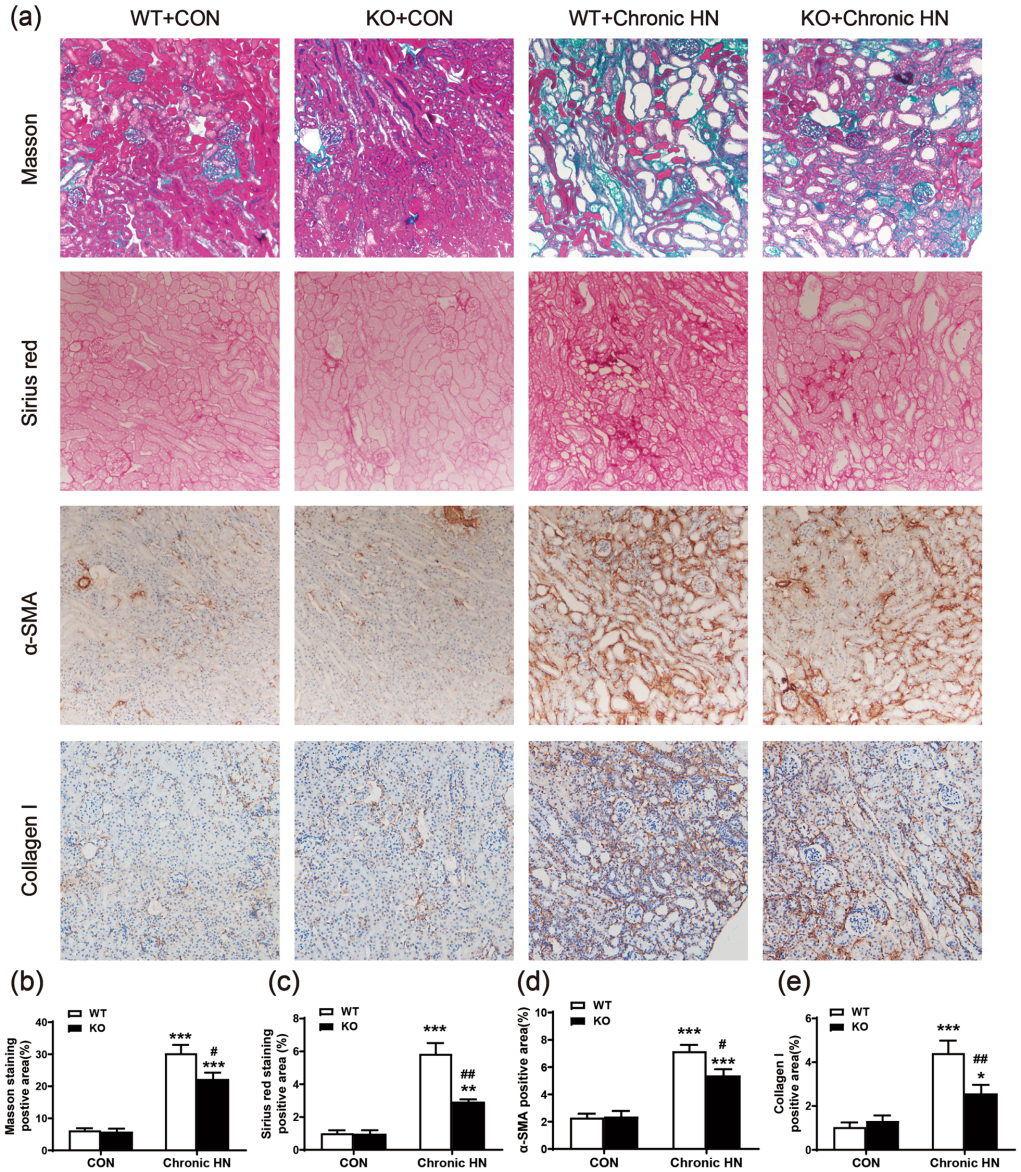
AMPK α2 exacerbated renal fibrosis in a mouse model of chronic HN. **a** Representative images of Masson staining, Sirius red staining, α-SMA, and collagen I immunohistochemical staining. **b** Quantitative analysis of Masson staining-positive area in the kidneys. **c** Quantitative analysis of Sirius red staining-positive area in the kidneys. **d** Quantitative analysis of the α-SMA-positive area in the kidneys. **e** Quantitative analysis of collagen I-positive area in the kidneys. Each bar represents the mean ± SEM. Versus CON, *P < 0.05, **P < 0.01 and ***P < 0.001; versus WT, ^#^*P* < 0.05 and ^##^*P* < 0.01

**Fig. 4:**
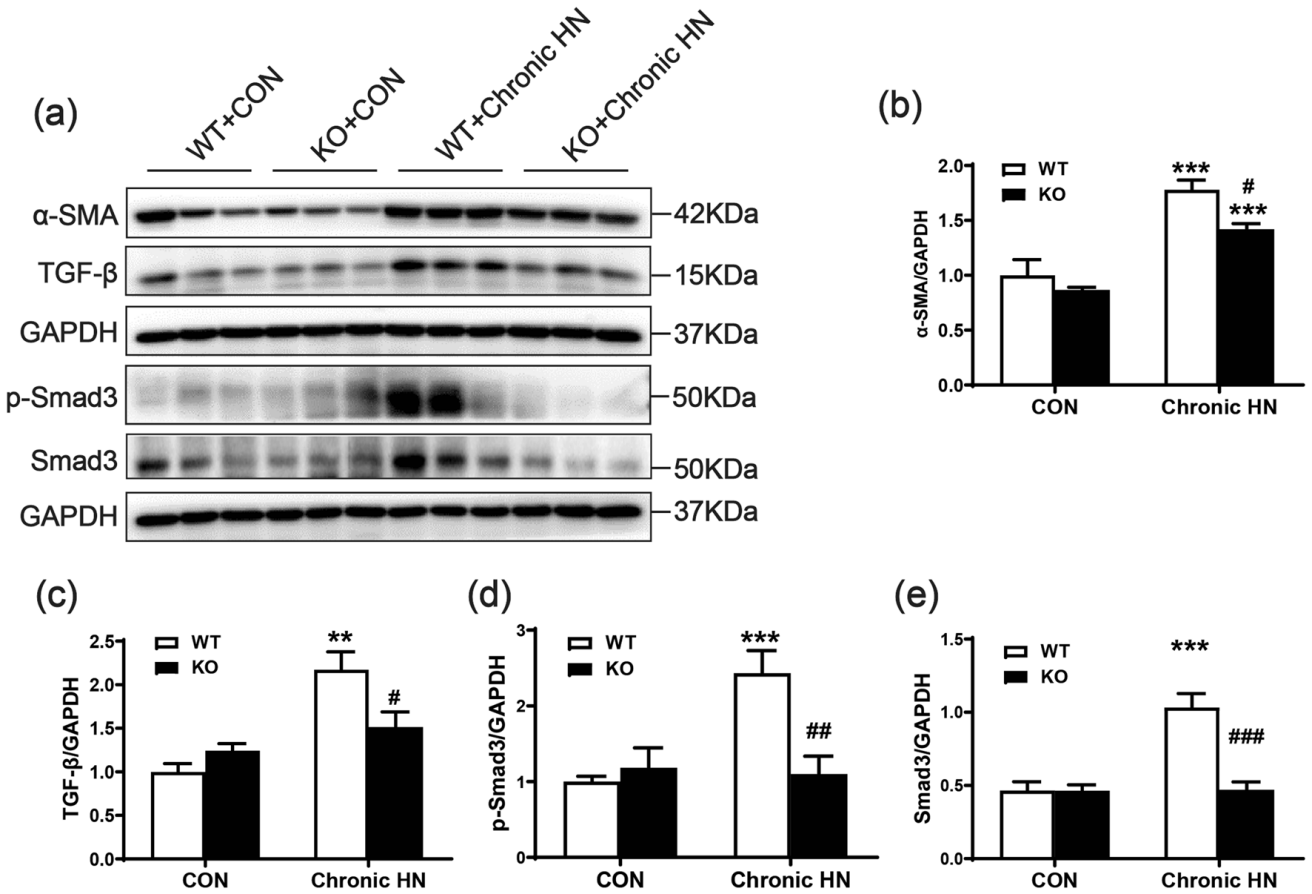
AMPK α2 exacerbated profibrotic TGF-β/Smad3 signaling in a mouse model of chronic HN. **a**–**e** Western blot analysis of α-SMA, TGF-β1, p-Smad3 and total Smad3 expression in the kidneys. Each bar represents the mean ± SEM. Versus CON, **P < 0.01 and ***P < 0.001; versus WT, ^#^*P* < 0.05 and ^##^*P* < 0.01

The role of AMPK α2 in renal fibrosis of chronic HN was next explored by Masson staining, Sirius red staining, and immunohistochemistry staining of collagen I. Elevated renal positive area of Masson staining and Sirius red staining in chronic HN mice indicated chronic hyperuricemia caused by renal fibrosis. In contrast, mice deficient in AMPK α2 developed less renal fibrosis (Fig. 3a–c). These findings were further confirmed by immunohistochemistry staining of collagen I (Fig. 3a and e). The expression of α-SMA, one of the markers of epithelial–mesenchymal transition (EMT), was also increased in chronic HN mice, whereas it was significantly downregulated in the chronic HN mice deficient in AMPK α2 (Figs. 3a and d; 4a and b). In addition, the expression of profibrotic cytokine TGF-β1 and its downstream p-Smad3 were also increased in chronic HN mice, as shown in Fig. 4, but it was significantly suppressed in chronic HN mice deficient in AMPK α2.

**Fig. 5:**
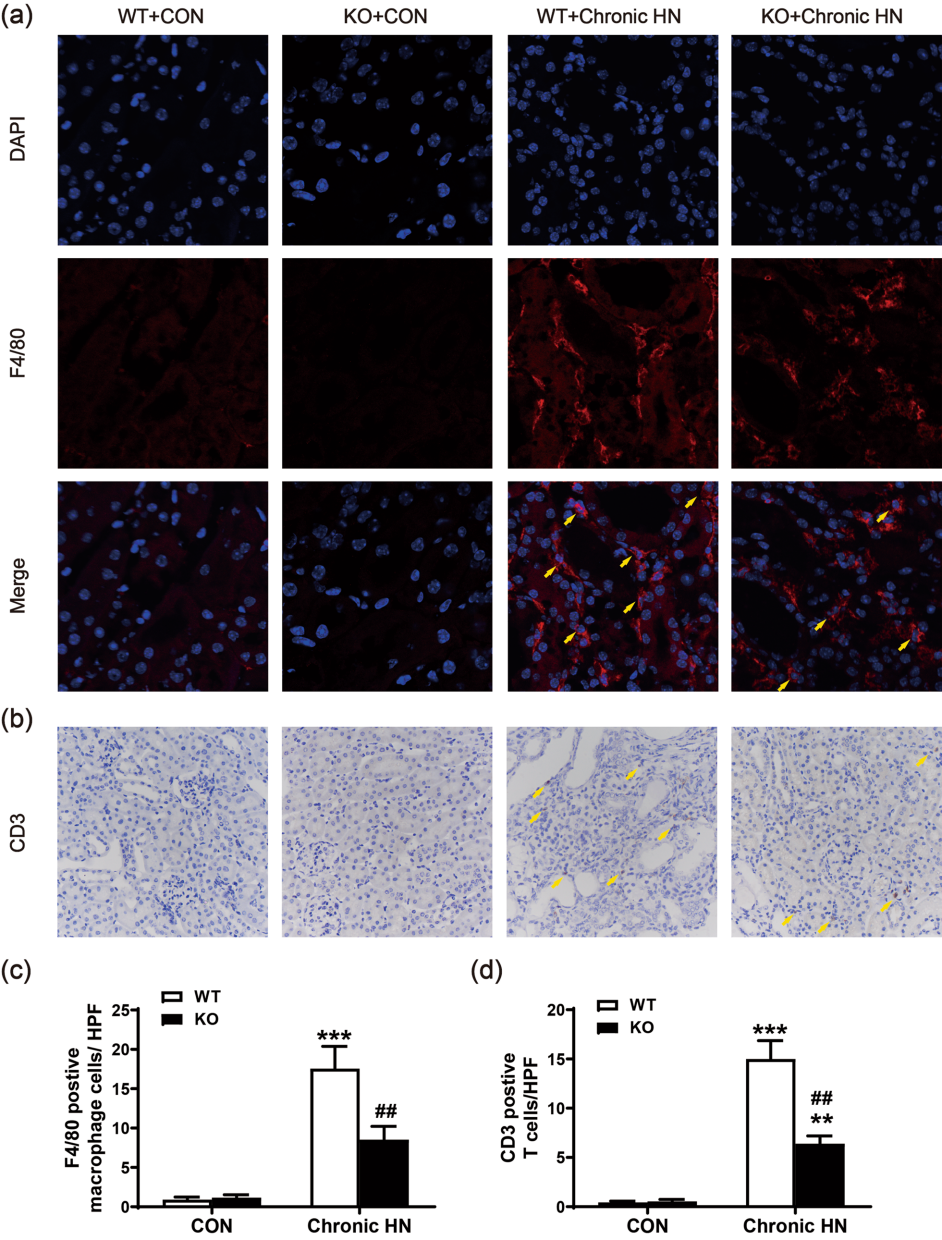
AMPK α2 exacerbated renal infiltration of proinflammatory cells in a mouse model of chronic HN. **a** Representative immunofluorescence images of F4/80-positive macrophages in the kidneys by a confocal microscope, red color indicates positive staining of F4/80, blue color stained by DAPI indicates nuclei. **b** Representative images of CD3-positive T cells in the kidneys by immunohistochemical staining. **c** Quantitative analysis of F4/80-positive macrophages in the kidneys. **d** Quantitative analysis of CD3-positive T cells in the kidneys. Each bar represents the mean ± SEM. Versus CON, **P < 0.01 and ***P < 0.001; versus WT, ^##^*P* < 0.01

Immunofluorescence of F4/80 and immunohistochemistry staining of CD3 was performed to determine the effect of AMPK α2 knockout on proinflammatory cell infiltration in chronic HN mice. The renal infiltration of F4/80-positive macrophages and CD3-positive T cells were significantly increased in chronic HN models, but were notably downregulated when AMPK α2 was knocked out (Fig. 5).

### AMPK α2 promoted renal urate crystal deposition in a mouse model of chronic HN

**Fig. 6:**
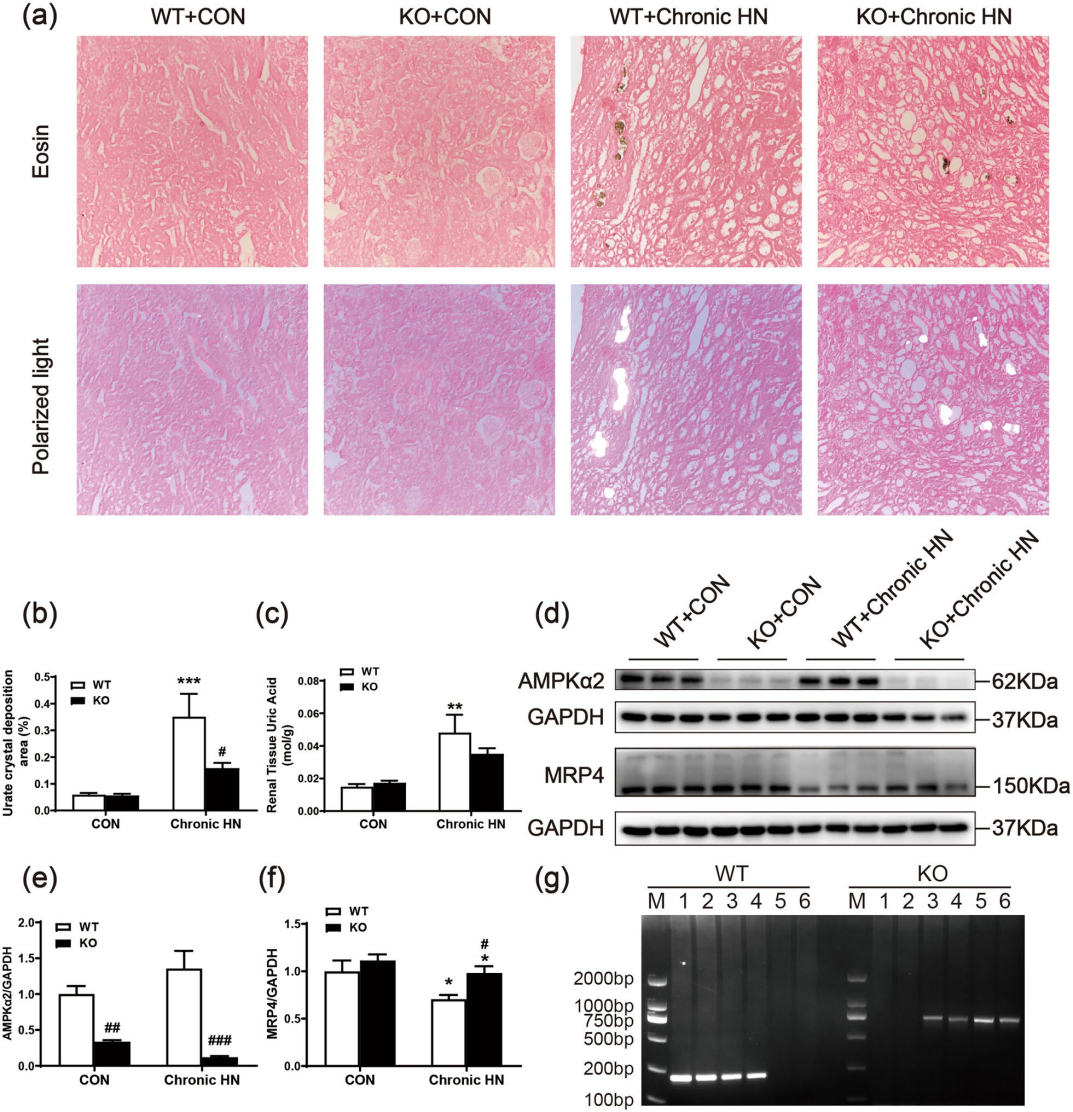
AMPK α2 promoted renal urate crystal deposition in a mouse model of chronic HN. **a** Representative images of eosin staining and compensation polarization microscopy of the entire anhydrous ethanol-treated kidneys. **b** Quantitative analysis of urate crystal deposition area in the kidneys. **c** Uric acid concentration per unit of renal tissue protein concentration. **d**–**f** Western blot analysis of AMPK α2 and MRP4 expression in the kidneys. **g** Genotyping of AMPK α2 knockout mice. M: DNA marker; 1, 2, wild-type homozygote, only DNA band near the 200 bp; 3, 4, heterozygote, DNA band of near both 200 bp and 600 bp; 5, 6, knockout homozygote, only DNA band near 600 bp. Each bar represents the mean ± SEM. Versus CON, *P < 0.05 and ***P < 0.001; versus WT, ^#^*P* < 0.05, ^##^*P* < 0.01 and ^###^*P* < 0.001

It is well accepted that the deposition of monosodium urate crystals in TECs and the interstitium results in renal damage. To explore the pathomechanisms of AMPK α2 in HN, eosin staining of the entire anhydrous ethanol-treated kidney specimens and semi-quantitative analysis of urate deposition in the kidneys was performed and detected by compensation polarization microscopy. In chronic HN mice, the insoluble urate crystal was mainly deposited in renal tubules, which were yellow in eosin-stained sections, and had a characteristic birefringence in compensation polarization microscopy (Fig. 6a and b). Compared with wild-type chronic HN mice, the urate crystal deposition was significantly decreased in AMPK α2 knockout HN mice. The renal level of uric acid was elevated in wild-type chronic HN mice, while deletion of AMPK α2 slightly downregulated the level of uric acid in chronic HN mice (Fig. 6c). Moreover, we tried to explore the potential mechanism of AMPK α2 promoting renal urate crystal deposition. As urate crystal deposition is regulated by urate reabsorption transporter and urate excretion transporters. No significant changes associated with AMPK α2 were observed among them (data were not shown) except multidrug resistance protein 4 (MRP4), an ATP-dependent UA transporter. Western blotting results showed that the relative expression of MRP4 was higher in chronic HN mice than in control mice. Knockout of AMPK α2 further upregulated the relative expression of MRP4 (Fig. 6d–g). These data suggested that AMPK α2 promoted renal urate crystal deposition probably through suppressing MRP4-mediated urate secretion.

**Fig. 7:**
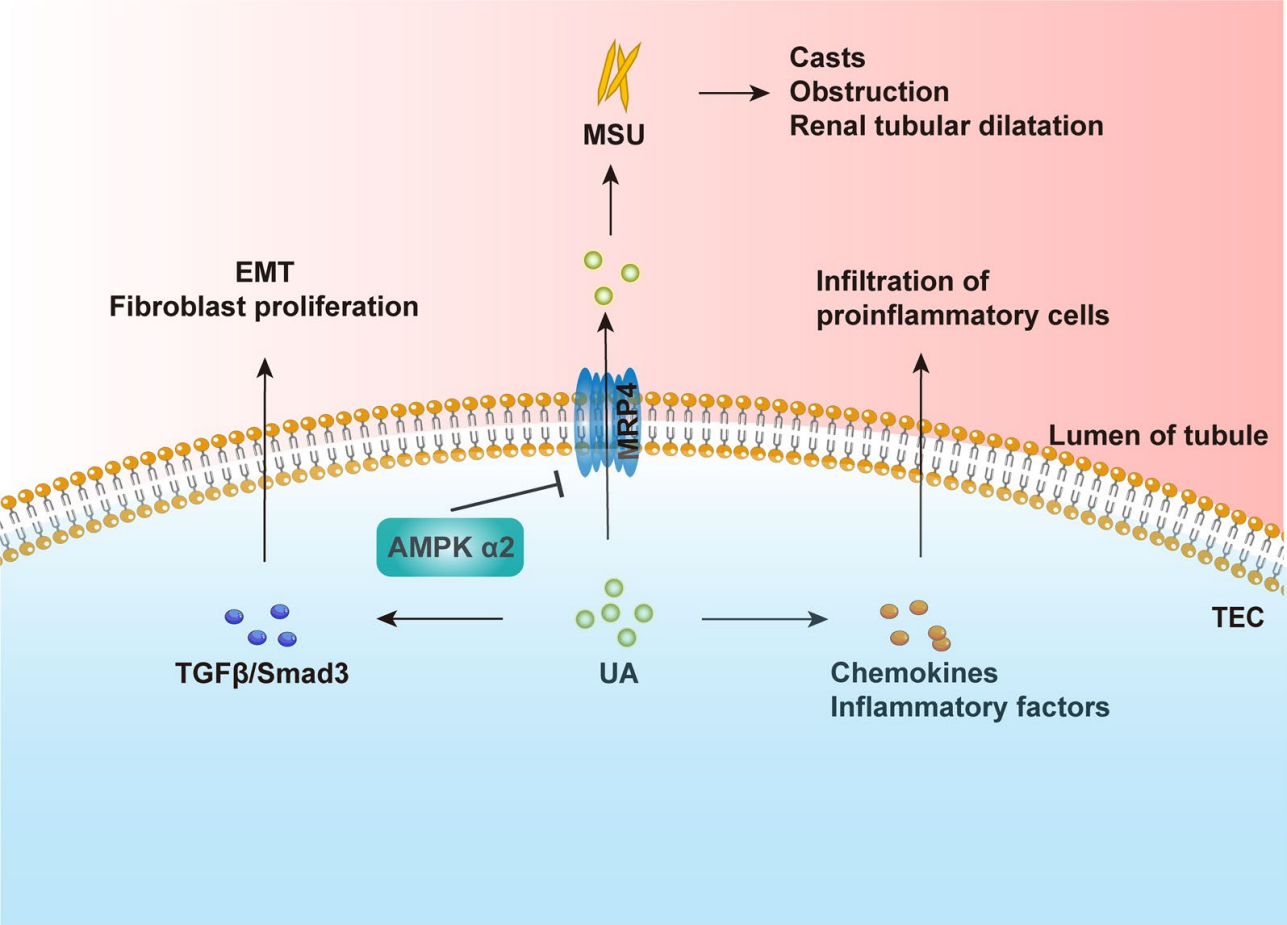
Schematic representation of AMPK α2 contributing to acute and chronic hyperuricemic nephropathy. AMPK α2 suppresses uric acid excretion through uric acid transporters. Accumulation of uric acid increased uric acid deposition, which further leads to the formation of a renal small tube type, inflammatory cell infiltration, and renal fibrosis

## Discussion

In this study, we examined the role of AMPK α2 in HN models. We produced classic acute and chronic HN mouse models by administering intraperitoneal injections with UA and OA in AMPK α2^+/+^ and AMPK α2^−/−^ mice. Our results have shown that AMPK α2 may contribute to renal injury and functional decline in HN models through the promotion of urate crystal deposition-induced inflammation and fibrosis (Fig. 7).

In the cast of long-term hyperuricemia, a large number of monosodium urate crystals (MSU) were deposited in the renal tubular lumen, renal pelvis, and ureter, leading to the formation of cysts, obstruction, and renal tubular dilatation, as well as the decrease of glomerular filtration rate and the increase of serum creatinine. The injured TECs and endothelial cells secrete various chemokines and inflammatory factors, mediating the infiltration of neutrophils, macrophages, dendritic cells, T cells, and other immune cells. Uric acid not only induces TEC injury, but also promotes NLRP3 inflammasome-dependent IL-1β, and IL-18 release in infiltrated macrophages and T cells, finally leading to renal inflammation. Aside from that, UA also stimulates the proliferation of T cells. Interestingly, AMPK α2 knockout suppressed the renal infiltration of proinflammatory macrophages and CD3-positive T cells, due to the downregulation of urate crystal deposition and TEC injury. In addition, chronic urate deposition in the tubular lumen of the kidneys also induces renal fibrosis via EMT and fibroblast proliferation. However, AMPK α2 knockout inhibited TGF-β/Smad3-mediated EMT and extracellular matrix production, due to the downregulated urate crystal deposition.

Impaired renal excretion of uric acid contributes to renal urate crystal deposition. Urate is reabsorbed via transporters including urate anion transporter 1 (URAT1), uric acid salt organic anion transporters 4 (OAT4), and glucose transporter protein (GLUT9). In contrast, other transporters, including OAT1, OAT3, urate transporters (UAT), multidrug resistance protein (MRP4/ABCC4), 4 ABCG2, and sodium-dependent phosphate transport protein, mediate urate excretion to lower blood levels of UA [22]. Alteration of these urate transporters in renal TECs is closely associated with hyperuricemia. In the present study, renal AMPK and AMPKα2 were activated in HN mice, while knockout of AMPKα2 notably suppressed total AMPK activation. Meanwhile, the renal expression of MRP4 has further increased in AMPK α2 knockout HN mice. These results indicate that AMPK activation inhibited urate execration maybe via down-regulating MRP4. It is well accepted that MRP4 is an ATP-dependent uric acid transporter that drains UA into the renal tubules by consuming energy. Also, AMPK plays a key role as a metabolic sensing regulator for multiple transport processes in the kidney [4, 14]. In particular, AMPK activation is expected to maintain cellular energy homeostasis by regulating renal epithelial ion transport in the absence of energy. In general, AMPK turns on energy production pathways (e.g., glucose uptake, glycolysis, fatty acid oxidation) and stops energy consumption processes (e.g., fat production, glycogen generation) to help cells survive at low energy levels [13]. Bataille AM et al. found that AMPK activation decreased urate secretion under cellular stress, and AMPK inhibitor compound C can prevent this effect [2]. Our findings provided new evidence in mouse HN models to support the previous study [2]. Unfortunately, due to the limited length of this article, we have not analyzed whether suppression of renal urate crystal deposition caused by AMPK α2 deletion is abrogated when MRP4 inhibition. The specific mechanism by which AMPK α2 regulates MRP4 remains unclear. MRP4 may be phosphorylated directly by AMPK α2 and then be ubiquitinated and degraded. AMPK α2 may also regulate target gene expression at the transcriptional level indirectly, as them have been discussed previously [2]. In this study, we used a mouse model in which only the AMPK α2 catalytic subunit was knocked out. AMPK α2 catalytic subunit mediates some part and not the full function of AMPK, hence, this was a potential limitation of the study.

## Conclusions

AMPK α2 contributed to acute and chronic hyperuricemia-induced kidney injury through the promotion of urate deposition. Further studies are still needed to explore the functions of catalytic subunits of AMPK in the urate reabsorption and execration in HN.

## Data Availability

The data that support the findings of this study are available from the corresponding author upon reasonable request.
